# Shame and STIs: An Exploration of Emerging Adult Students’ Felt Shame and Stigma towards Getting Tested for and Disclosing Sexually Transmitted Infections

**DOI:** 10.3390/ijerph18137179

**Published:** 2021-07-05

**Authors:** Emily Scheinfeld

**Affiliations:** School of Communication and Media, Kennesaw State University, Kennesaw, GA 30144, USA; escheinf@kennesaw.edu; Tel.: +1-470-578-2572

**Keywords:** sex communication, disclosure, shame, stigma, emerging adults, sexually transmitted infections (STIs), parent–child communication

## Abstract

Emerging adulthood is identified as a time of identity exploration, during which emerging adults (EAs) may engage in sexual exploration and risky behaviors, potentially resulting in the contraction of a sexually transmitted infection (STI). Many EAs, do not disclose their status to partners or those who can provide social support, like parents. Nor do they often get tested. This may be due to the changing status of stigma surrounding STIs. This study examines traditional measures of the stigma/shame of STI diagnoses, treatment, and testing, and their relevance alongside both increased opportunities for casual sex and not only heightened education surrounding STIs, but also heightened prevalence of STIs in the U.S. Results show EAs perceived that if their community found out they got tested, they would likely be treated differently. They also felt they would be uncomfortable disclosing an STI to parents as well as to sexual partners. However, disclosing to a monogamous partner yielded less felt shame and stigma by EAs. Lastly, stigma/shame was associated with STI communication, as well as with overall perception of STI knowledge, and getting tested. Further explanation of the results and possible implications of this study are discussed.

## 1. Introduction

Sexually transmitted infections (STIs) are a growing issue in the United States, with over 26 million new cases in 2018 alone [1]. Emerging adults (EAs; age 18–25) comprise 25 percent of the sexually active adult population and yet they make up 50 percent of all new STI cases [2]. Emerging adulthood is a transitional time, often marked by personal and sexual exploration [3] and the development of personal health behaviors [4] as adolescents navigate their way into adulthood. During this period, EAs are more likely to partake in “risky” sexual behavior, such as having multiple and simultaneous sexual partners and using protection less frequently [5] perpetuating the spread of STIs [6,7]. Additionally, the Centers for Disease Control and Prevention (CDC) has noted that EAs’ lack of STI screening increases their risk of contraction [1]. Several demographic characteristics may lead to this behavior [8], including a sense of invulnerability and perceived barriers to testing. 

The low testing rates among emerging adults, and low levels of disclosure have been attributed in part to shame and stigma [8]. However, some research has found a lack of shame or stigma toward STIs in general, talking about STI testing with health care providers, and getting tested [9,10]. Perceived shame (the expectation of society’s negative reaction) and stigma (the perception of failing to meet social standards) involve the culture an individual is part of. The lack of these reactions may be due to a variety of factors, including the “hook-up culture” among this demographic, specifically on college campuses [11], increased sex education, and/or the perception that STIs are treatable, if not curable [1]. There has also been increased awareness surrounding HPV and its prevalence in recent years [2]. Although this is concentrated on one specific STI, such awareness may decrease the shame and stigma surrounding the medical and communicative aspect (specifically talking to health care providers) of STIs as a group.

Thus, unwillingness to get tested may not come from felt shame and stigma of the STI. Despite many EAs engaging in, or being surrounded by, a “hook-up culture” [11], this demographic remains uneasy when talking about sexual behaviors, sexuality, and STIs to sexual partners and/or parents when seeking support. Noar and colleagues [12] argue that open communication can promote safe sex practices and healthier decision making. However, sex communication, condom negotiation, and STI disclosure are considered by many to be daunting and uncomfortable tasks. Therefore, the aim of this study is to delve into sex communication between EAs and their parents, monogamous sexual partners, and casual sexual partners, as the shame and stigma may potentially be more closely related to the process of engaging in conversation about STIs and disclosing an STI status than the STI itself.

## 2. Literature Review 

### 2.1. Emerging Adults 

Arnett [13,14] argues that continued reliance on parental figures, in conjunction with falling within the age range of 18–25, defines an individual as an emerging adult. Oftentimes this dependency is connected to the notion that EAs are prolonging their time until full adulthood and are attending college longer, and thus in need of financial support [14].

Relevant to this study, EAs also continue their dependence on parents’ health insurance plans. The onset of the Affordable Care Act (ACA) allows EAs to remain under their parents’ health insurance through 26 years of age. Immediately following the approval of the ACA, more than three million EAs who did not have coverage before 2010 were able to get medical coverage on their parents’ plan [15]. EAs have the highest rate of uninsured than any age group at 30% and have the lowest rate of access to employer-based insurance as they transition into the work force in entry-level and part-time jobs, or even take positions with small businesses unable to provide employer-sponsored insurance [16]. Thus, many EAs remain on their parents’ health insurance plans as long as they can due to this lack of access, as well as the lower or no cost to them, since many parents pay the premium [17].

By even subjective definitions [13], these 18–26 year olds remain steadfast within emerging adulthood. As these EAs increase the amount of financial dependence on parents, they may also be welcoming more parental involvement, specifically in times of illness or injury. That is, continued reliance on parents during emerging adulthood blurs boundaries of what information may be deemed private, and when that information is then financially supported by parents (e.g., getting parent-paid health insurance to cover STI tests or treatments), the boundaries around private information, even those around sensitive and highly personal facts, go completely out of focus. Thus, although EAs desire more restrictive boundaries around their personal and private information, like sexual behaviors, continued reliance financially and through insurance makes privacy boundaries difficult to navigate [18]. Moreover, parents may realize the importance of refraining from interfering, but providing support can be perceived by parents as permission to co-own personal details, including problematic behaviors and sexual activity [19]. On the other hand, EAs continue to feel tension [18] surrounding their relationship with their parents as it transforms during the emerging adulthood years. EAs may feel obligated to disclose sensitive information like sexual activity to receive support and/or create intimacy with a parent, and yet also want to withhold personally private information to gain, or maintain, autonomy and independence [18].

### 2.2. STI-Related Shame and Stigma

Shame and stigma have the potential to discourage STI testing and treatment. Health-related stigma refers to the “expectations of society’s negative reaction to the health condition” [20], potentially resulting in discrimination because of a particular trait. Shame is a more internally motivated reaction, defined as “a negative emotion elicited when a person experiences failure in relation to a personal or social standard, feels responsible for this failure, and believes that the failure reflects self-inadequacy” [8,21] Research has suggested that stigma and shame related to STIs are common in the U.S. [22]. Specifically, felt STI shame and stigma have also been tied to failure to test for STIs [21,23], delaying care [8,21], and discussing sexually related topics with a romantic partner or health care provider [24,25]. Morris et al. [22] continued this line of research, finding similar results among male African American low-income youth, where stigma was a greater indicator of avoiding treatment and testing than shame [22]. However, much of the research remains within specific demographics [22,26,27].

Past research using traditional measures of assessing shame and stigma towards STIs (i.e., assessing treatment, health support seeking, and testing) have shown sufficient variance of stigma and shame related to STIs [28]. However, in more recent studies, the measure for shame and stigma towards STIs remained skewed to the left from study to study [9,10]. That is, most of the participants of these studies experienced less shame or stigma towards STIs on average. This may be due to a variety of factors, including increased sex education and understanding of the infections. Many STIs are now treatable, allowing the infection to subside [1,29]. There is also increased knowledge that infections such as HPV—despite being dangerous, causing various forms of cancer, if the immune system is unable to protect itself against the virus—often go unnoticed by the individual and can disappear over time [2]. The CDC [2] indicates that over fifty percent of sexually active individuals in the United States are infected with HPV at some point in their life. Such prevalence, lack of symptoms, and ease with which to treat may decrease the shame and stigma surrounding STIs. 

The culture surrounding sex during emerging adulthood may have also decreased the role of shame and stigma. EAs may be more exploratory than established adults when it comes to risky behaviors, including sex [3]. Additionally, EAs are increasingly partaking in the hook-up culture, and engaging in sexual behaviors outside of committed relationships [30]. This behavior is widely accepted and typical on many college campuses [30,31], which could indicate that STIs are less of a concern than more pressing social issues (e.g., to be perceived as casual about sex). However, the hook-up culture does compound the risk of infection. Those who hook up are more likely to have multiple and simultaneous sexual partners [5] and are less likely to use, or ask for, protection [7]. Breaking these “hook-up norms” is stigmatized, potentially more than the STIs that come of it, despite the widespread dissatisfaction with them [31].

The traditional measure of STI shame and stigma [8] is to assess the stigma of getting tested for STIs and the perception of individuals who test positive. Due to the hook-up culture and increased STI education among EAs, such a measure may no longer be sufficient. That is, if EAs are better able to manage their impression [32] in a health care setting when seeking testing or treatment, the shame and stigma of an STI may be more readily observed in other communicative processes. For example, this demographic has been shown to experience uneasiness when talking about sexual behaviors, sexuality, and STIs with those close to them. In fact, despite widespread understanding that open communication about sexual behaviors can promote safer sexual decision-making [12,33], sex communication (e.g., asking questions, disclosure of STIs, asking for help) with parents and sexual partners remains limited in nature due to being condemned as taboo, uncomfortable, and daunting. 

### 2.3. Shame Resilience Theory

Brown argues that shame, and thus similarly, stigma, is a painful experience or feeling coming from the idea that we are flawed or unworthy for some reason [34]. STIs have long been categorized as health issues that not only encourage people to discredit others, but also to feeling discredited themselves following an STI diagnosis. Goffman explains that stigma is deeply discrediting [35]; thus, as shame and stigma are social and communicative by nature [36], people feel discredited by others judging or discriminating them due to their stigmatizing flaw. Charmaz elaborates on these ideas of shame and stigma since illness makes people vulnerable to negative social identifications, but also their own self-definitions [37]. They feel discredit-*able*, and that is enough for them to perceive felt shame and stigma. 

Brown proposes that people can become resilient to shame and stigma by decreasing their own feelings of being trapped, powerless, and isolated [34]. She continues that resilience comes with an increase in experiencing empathy and connection with others [35], an inherently communicative process. This connection allows for people of similar identities to have “mutual support, shared experiences, and the freedom to explore and create options” [34]. Although not all EAs are diagnosed with an STI, it is possible that EAs are already connected to one another by not only being of a similar demographic (i.e., similar age range, reliance on parents, in school, etc.), but also being a part of the hook-up culture. Shame and stigma literature usually examines how people cope with and communicate about their perceived experiences when it comes to a diagnosis, disability, skin color, or religion. However, potentially being young and seemingly promiscuous is stigmatizing in and of itself, and others stigmatize them based on this supposed blemish of character (e.g., promiscuous sex). Based on shame resilience theory [34], EAs may recognize this vulnerability to the “outside world,” form connections, are somewhat isolated on college campuses, and are empathetic of the outcomes of their behaviors, including those that may cause more stigmatizing identities, like an STI diagnosis. In other words, EAs may preemptively be resilient to STI shame and stigma due to the resilience they developed as a coping mechanism to deal with older adults’ stigmatization of their college lifestyle. Thus, this resilience allows EAs to face other shame and stigma traditionally associated with additional stressful events, like disclosing an STI diagnosis or getting tested. 

### 2.4. Research Questions

Research surrounding sex communication has focused on the notion and the extent to which it is uncomfortable [38], as well as its effect on behavior [39]. Conversely, research concerning STIs focuses on the outcomes of not disclosing: avoiding disclosure altogether and/or avoiding getting tested [21,23]. Thus, there is a gap in the literature regarding the shame and stigma associated with the idea of sex communication, conversations surrounding safe sex with sexual partners, and the disclosure of STIs. Disclosure is both critical and complicated. A meta-analysis emphasized the positive outcomes of disclosing, including the positive relationship to social support, self-competence, reduced anxiety, and decreased problem behavior [40]. In terms of deciding to disclose, this study found that timing, opportunity, cognitive ability of the receiver, preparation of the message, and advantages and disadvantages of disclosure all affect the decision. 

Researchers are aware that there is shame and stigma associated with STIs but have not yet examined whether there is shame and stigma associated with the act of disclosing a status within interpersonal relationships, or the variables surrounding the communicative process. Moreover, given the lack of research in shame resilience theory in the context of STIs, it is unknown whether EAs have in fact developed a level of resilience to shame and stigma surrounding their lifestyle, which could include STIs, nor is it understood whether the receiver of the disclosure impacts the resilience or felt shame and stigma. Research [40] also does not address how shame and stigma confound other processes like disclosure (e.g., revealing sexual activity to a parent). or other related communicative interactions around sex (e.g., safe sex talks). Therefore, although research has been conducted on the role of stigma on delaying STI treatment and care, little research has delved into the stigma and shame surrounding disclosure of an STI, and the following research questions are posed:

*RQ1*: How do EAs perceive they will be treated by others if specific others find out they have been (a) tested for STIs; and (b) the results of those STI tests?

*RQ2*: Are there differences in the perceived shame and stigma associated with disclosing a STI to a (a) parent; (b) monogamous sexual partner; or (c) casual sexual partner?

*RQ3*: How do the shame and stigma related to STIs impact communicative processes, like communication about STIs, disclosing an STI diagnosis, and sex communication? 

*RQ4*: What is the relationship between shame and stigma related to STIs and EAs’ perception of their knowledge surrounding STIs? Additionally, how does that impact communication with others?

## 3. Materials and Methods

### 3.1. Participants

Participants included 462 undergraduate students, of ages 18–25 (M = 20.09; SD = 1.29), from a large American Southwestern university. Undergraduate students were chosen to represent the EA population because approximately half of adults between 18 to 24 have enrolled in or have completed college studies [41], and the college hook-up culture was also a contributing factor within this study. Another motivation was the ability to employ convenience sampling, which in this case allowed for lowered cost and research time and a high participant turnout. Students were recruited through announcements in their courses for extra credit. In order to participate, they must have been between the ages of 18 and 25 years, which is classified as the emerging adulthood stage [13,14]. The majority of the participants were female (*n* = 359; 80.7%). Over half of the sample identified as non-Hispanic and White (*n* = 276; 62%) followed by Hispanic or Latino (*n* = 57; 12.8%). Demographic characteristics and subsequent analyses are based on 444 of the participants, as 18 did not complete several measures. The majority of the participants were sexually active, with over half (*n* = 350; 78.7%%) having engaged in vaginal sex; 86.7% (*n* =386) indicated having received or given oral sex. There was an even distribution of single (*n* = 178; 40.0%) participants and those who identified as being in a monogamous, long-term relationship (*n* = 197; 44.3%). Some claimed to be in casual relationships or dating multiple people at the same time (*n* = 64; 14.4%), while one participant indicated s/he was married and another that s/he was divorced. Lastly, well over half of the participants indicated they belonged to some religious organization (*n* = 276; 62.0%), identifying primarily as Catholic (*n* = 71; 16.0%), Baptist or Methodist (*n* = 66; 14.8%), or non-denominational Christian (*n* = 72; 16.2%). Relatedly, approximately half of these individuals are also engaging in religiously affiliated activities at least once a week (*n* = 143; 32.2%). This is consistent with the religious demographics for the geographical area. 

### 3.2. Procedures

Recruitment emails and website posts included a link to the survey, hosted online by Qualtrics. After completing a consent form, the first portion of the questionnaire included questions pertaining to participants’ past sexual behaviors and safe sex practices. Participants then completed a short health literacy measure, as well as an STI knowledge quiz. Using a variety of scale measures, participants were asked to report on their ability to communicate about STIs, as well as their perception of how others would treat and/or support them if others knew they were getting tested for, or had contracted, an STI. Participants’ shame and stigma was assessed to examine their feelings towards STI diagnoses, getting tested for STIs, and disclosing a hypothetical status to parents, romantic monogamous partner, or casual partner. Lastly, demographic information was collected, including age, sex, religious affiliation, relationship status, health insurance coverage, and current living situation (i.e., whether they live in a dorm, apartment, or home, and whether they have roommates). Before exiting the site, participants were debriefed of this study’s goals. As this study is part of a larger data set, the full survey took approximately 20 min, depending on the pace of the participant. 

### 3.3. Measures 

#### 3.3.1. Perception of Treatment from Others 

This measure was adapted from an assessment tool developed by Pandya and colleagues [42] to assess perceived impact of disclosing a mental illness. Participants were asked to indicate whether they thought they would be treated worse, better, or not differently by specific groups of people following certain disclosures. First, they indicated treatment when the other party knew they had gotten tested, and then if the other party had found out positive results of the test. As per Pandya et al. [42], groups included police, employer, co-workers, extended family, roommates, health care providers, friends, partners, parents, and places of worship. 

#### 3.3.2. Perception of Available Social and Medical Support 

As with disclosure experiences in the previous section, those who received the first-person questionnaire were only asked to respond to this measure if they indicated they had ever been diagnosed with an STI. Additionally, adapted from Pandya et al. [42] to address STI status rather than mental illness, this measure had participants use a 7-point Likert scale (1 = strongly disagree; 7 = strongly agree) to indicate how much they agreed with eight statements pertaining to their ability to rely on health care providers and the health care system, family, friends, and partners. For example, “I know I can rely on my parents, siblings, or extended family to help me when I need it,” or “People I relied on became distant after they learned I’d been diagnosed with an STI.” Reliability was adequate (*α* = 0.71; *M* = 3.77; *SD* = 0.65). 

#### 3.3.3. Willingness to Disclose of STI Status 

Participants were first asked “if they had been, or were to be, diagnosed with an STI, would they disclose this information” to their parent, monogamous partner, or casual sexual partner. If they indicated no, they were then asked to use a 7-point Likert scale (1 = strongly disagree; 7 = strongly agree) to indicate how much they agreed with three items asking why they did or would not disclose an STI status: “I do not want to burden my parent(s) with the knowledge that I had contracted an STI,” “I did not think it was important to disclose to my parent(s),” and “I consider my STI status a private matter.” For the scale asking about disclosure to parents, participants also responded to the item, “I do not keep in touch with my parent(s).” This phrasing and method were used when asking about disclosing to a parent (*α* = 0.61; *M* = 3.78; *SD* = 0.80), a monogamous romantic partner (*α* = 0.81; *M* = 2.86; *SD* = 1.07), and a casual sexual partner (*α* = 0.76; *M* = 3.17; *SD* = 0.92). This measure was adapted from a tool developed by Aktan-Collan et al. [43] to assess disclosing genetic risk with family members. The subject matter was the primary change, but the receiver of the disclosure was also adjusted to include all three potential confidants indicated above. 

#### 3.3.4. Communication about Sex and STIs 

Twenty items were used to assess communication about sex, tapping into the general attitudes and experiences participants had had talking about sex with their parents and their romantic and/or sexual partners [44]. Eight were specific to talking with parents about sex (e.g., “My parent and I have discussed abstinence as a way of preventing STIs”), while the remaining twelve addressed communication with dating or sexual partners (e.g., “I have discussed the use of condoms with my partner as a STIs prevention method”). Participants were asked to indicate how much they agreed or disagreed with each of the statements using a 7-point Likert-type scale (1 = strongly disagree; 7 = strongly agree). Consistent with past research, the parent version was internally consistent (*α* = 0.94; *M* = 2.80; *SD* = 1.12). After running a principal axis factor analysis with varimax rotation, the dating or sexual partner version yielded three factors or themes: general discussions about STIs with partners, speaking openly and honestly with partners, and abstinence as a form of protection. Item 12, “using a condom would be embarrassing” was removed due to low item-total correlation (0.04). Despite three factors with eigenvalues over one, the remaining eleven items had relatively high inter-item reliability (*α* = 0.90; *M* = 3.09; *SD* = 0.84). 

#### 3.3.5. Shame and Stigma towards STIs 

This scale assesses reactions to STIs and STI-related testing [8]. Using a 7-point Likert-type scale (1 = strongly disagree; 7 = strongly agree), participants responded to five items pertaining to stigma towards STIs (e.g., “Getting a sexually transmitted infection would make me feel lonely,” and “Getting a sexually transmitted infection means I have poor morals”). The other six items indicated a sense of contamination and shame towards an STI diagnosis (e.g., “people with sexually transmitted infections should be ashamed of themselves”). A principal axis factor analysis with varimax rotation indicated both factors were present: stigma and shame. There was high reliability amongst the items (*α* = 0.90; *M* = 2.57; *SD* = 0.76). 

#### 3.3.6. Shame and Stigma of Disclosing an STI 

Similar to the measure evaluating stigma towards STIs, a measure was developed based on the aforementioned [8] scale for the purpose of this study to examine EAs’ felt stigma towards disclosing an STI to parents, monogamous romantic partner, or casual sexual partner. Items were adapted from the above-mentioned measure to address each confidant. For example, “I would feel dirty to tell my parent I had been diagnosed with an STI,” or “If I told a romantic partner I had been diagnosed with an STI, they would assume I had poor morals.” Each of the measures were relatively high in reliability (parent: *α* = 0.92; *M* = 3.40; *SD* = 1.04; a monogamous romantic partner: *α* = 0.91; *M* = 3.38; *SD* = 0.90; a casual sexual partner: *α* = 0.91; *M* = 3.43; *SD* = 0.90). 

#### 3.3.7. Demographic Information 

Several additional data points were collected as possible covariates within the analysis, such as gender, age, and ethnicity. Participants were asked to indicate whether they were currently sexually active as well as their sexual health practices (e.g., condom usage and consistency). Participants were also asked about their current relationship status, insurance coverage, religious affiliation, religiosity (frequency of participating in religious events), current residence (e.g., dorm versus apartment), and whether they had roommates. 

## 4. Results

### 4.1. Preliminary Analyses

Descriptive analyses were conducted to examine the mean, standard deviation, and distribution of all primary variables. Unless otherwise noted, scales were checked for normality, unidimensionality through the use of exploratory factor analysis with varimax rotation. Although some of the variables showed more than one factor, tests for internal reliability displayed consistency and relatively good reliability. Therefore, an aggregate measure was created by summing the items and taking the mean to create a scale for each variable. Bivariate correlations between all aggregates and summed variables were also examined before testing the research questions (see Table 1).

To utilize all available data, expectation maximization (EM) was used. EM is a maximum likelihood procedure in which the parameters are estimated, then missing values are estimated [45,46]. Additionally, EM infers values based on the likelihood under the normal distribution and is advantageous as it produces nearly unbiased estimates of means, variances, and co-variances [46]. 

### 4.2. Perception of How Others May Treat EAs (RQ1)

**Figure 1:**
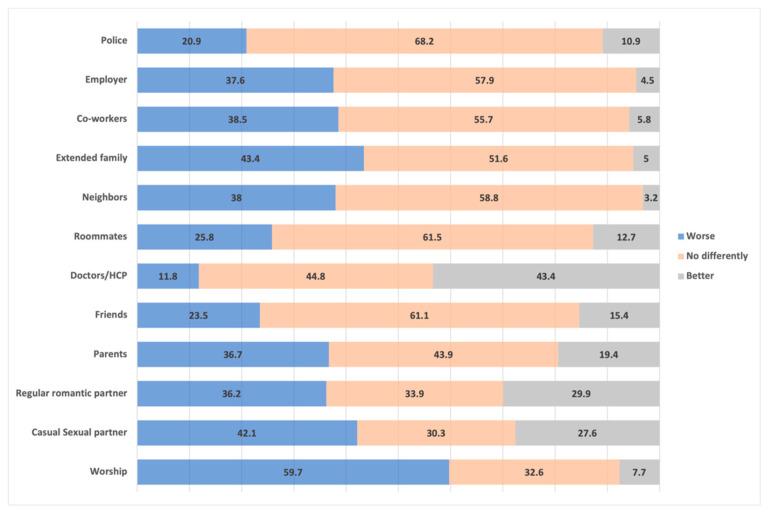
Percentage of participants who perceive they would be treated better, worse, or no differently by specific individuals or groups after disclosing they had been tested for an STI.

**Figure 2:**
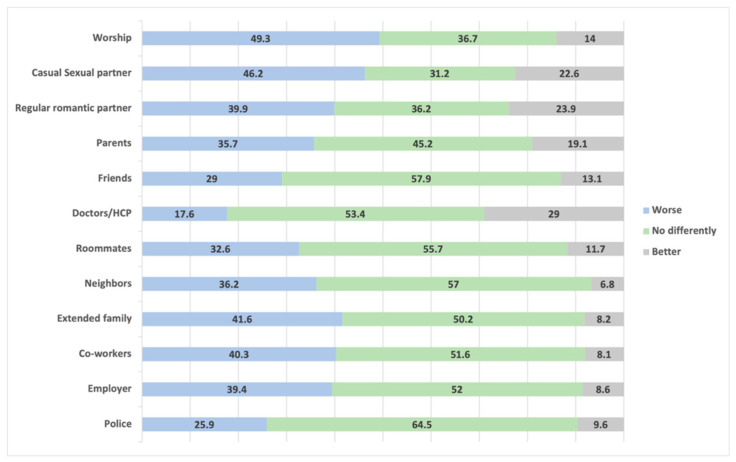
Percentage of subjects who perceive they would be treated better, worse, or no differently by specific individuals or groups after finding out the results of an STI test.

The first research question asks how EAs perceives others in their life and community may treat them if they find out they had been tested for an STI and if they found out the results of said tests. In most cases, EAs thought they would be treated no differently if loved ones or community members found out they had been tested for STIs (see Figure 1). Over half of the participants did perceive that those from their religious community may treat them worse (59.7%). Over 40% also perceived casual sexual partners and extended families may also treat them worse. The main difference between these two groups, however, is that 27.6% thought casual sexual partners may treat them better, compared to only 5% of extended family members (i.e., 51.6% of EAs perceived their family would treat them no differently). On the opposite end, EAs perceived doctors and other health care providers would likely treat them better (43.3% compared to 11.8% who thought their HCP would treat them worse). The outcomes were relatively similar if their loved ones and community members found out about STI results, with the majority of EAs perceiving most people would treat them no differently (see Figure 2). 

### 4.3. Associations between Disclosing to Parents, Monogamous, or Casual Partner (RQ2)

The second research question asked the differences between perceived shame and stigma associated with disclosing STIs to parents, monogamous sexual partners, and casual sexual partners. To explore this question, one-way repeated-measures ANOVA was used in order to test for significant differences between traditional forms of STI-related shame and stigma and the shame and stigma related to disclosing to a parent, monogamous romantic partner, or casual sexual partner. Mauchly’s Test indicated that the assumption of sphericity had been violated, *W* = 0.96, *χ*^2^ (2) = 20.17, *p* < 0.001, therefore degrees of freedom were corrected using Huynh–Feldt estimates of sphericity (*η* = 0.96). The results show there was a significant difference of perceived shame and stigma depending on who EAs were to disclose an STI status to, controlling for the traditional measure of STI shame and stigma, *F*(1.91, 840.20) = 4.74), *p* = 0.01. Specifically, at *p* < 0.001, there was a significant difference between perceived stigma and shame when disclosing an STI diagnosis to a monogamous partner in comparison to disclosing an STI diagnosis to parents, controlling for the traditional measure of general STI shame and stigma (Fortenberry et al., 2002). There were also significant differences between perceived shame and stigma when disclosing to a monogamous partner compared to a casual sexual partner. However, there were no significant differences between perceived shame and stigma of disclosing an STI status to parents compared to perceived shame and stigma of disclosing to a casual sexual partner (see Table 2).

### 4.4. Relationships between Shame and Stigma and Sex Communication (RQ3)

The third research question asked how STI-related shame and stigma impact other communicative processes, like communicating about STIs, actually disclosing an STI diagnosis, and safe sex communication. To explore RQ2, several regression analyses were conducted with shame and stigma pertaining to STIs being the independent variables, controlling for sexual experience (oral and vaginal), whether participants had been tested for an STI before, whether participants had tested positive for an STI before, and religiosity by placing the variables into Block 1. 

The first text placed the aggregate of shame and stigma into Block 2 and parent communication as the dependent variable. The overall model was significant (*F*(5,184) = 2.43, *p* = 0.04, adjusted *R*^2^ = 0.04, *R*^2^ change = 0.03), as was the standardized coefficient specific to STI-related shame and stigma on EA’s communication with parents about STIs (*ß* = −0.16, *p* = 0.03). Thus, the shame and stigma, along with the control variables, accounted for 6.2% of the variance; shame and stigma accounting for 2.5% of the variance on its own. The second test placed partner communication about STIs as the dependent variable. The overall model was once again significant (*F*(5,184) = 3.24, *p* = 0.008, adjusted *R*^2^ = 0.06, *R*^2^ change = 0.03), as was the standardized coefficient (*ß* = −0.19, *p* = 0.01). The independent variables accounted for 8.1% of the variance; specifically shame and stigma accounting for 3.4% on its own. Both of these relationships were negative, indicating the more shame and stigma surrounding STIs an EA perceives, the less likely they are to engage in conversation with their parents or any sexual partner about STIs.

This research question also digs into whether shame and stigma surrounding STIs impacts disclosing STI diagnoses to parents, monogamous romantic partners, and casual sexual partners, as well as to why EAs would or would not disclose that information. Running whether EAs disclosed (or would disclose given the necessity) an STI status to their parents on shame and stigma surrounding STIs yielded a non-significant overall model (*F*(5,85) = 0.75 *p* = 0.59, adjusted *R*^2^ = −0.01, *R*^2^ change = 0.004). The test running why they would or would not disclose an STI diagnosis was also non-significant (*F*(5,32) = 0.42, *p* = 0.83, adjusted *R*^2^ = −0.09, *R*^2^ change = 0.004). Similarly, the relationship between perceived STI shame and stigma and disclosing to a monogamous romantic partner was non-significant (*F*(5,86) = 1.50, *p* = 0.20, adjusted *R^2^* = 0.03, *R^2^* change = 0.002). Lastly, the relationship between perceived STI shame and stigma and whether EAs would or would not disclose and STI to a casual sexual partner was significant (*F*(5,86) = 2.48, *p* = 0.03, adjusted *R*^2^ = 0.08, *R*^2^ change = 0.01), but the standardized coefficient was not (*ß* = 0.07, *p* = 0.54), indicating that the specific independent variable of shame and stigma was not significantly associated, as it only accounted for 1% of the variance. 

### 4.5. Partial Mediations (RQ4)

The last research question digs into EAs’ perceived knowledge of STIs and how that impacts not only their perceived shame and stigma surrounding STIs, but whether they get tested for STIs, as well as how these issues in turn impact communication with others. A regression analysis controlling for sexual experience (oral and vaginal), whether participants had tested positive for an STI before, and religiosity by placing the variables into Block 1. Knowledge perception of STIs was placed into Block 2, and the shame and stigma aggregate was entered as the dependent variable. The overall model was significant (*F*(5,86) = 2.70, *p* = 0.20, adjusted *R*^2^ = 0.09, *R*^2^ change = 0.05), as was the standardized coefficient (*ß* = −0.22, *p* = 0.04). The overall model regressing getting tested for STIs on perceived knowledge was significant (*F*(5,86) = 25.30, *p* < 0.001, adjusted *R*^2^ = 0.19, *R*^2^ change = 0.02), but the standardized coefficient was not (*ß* = −0.14, *p* = 0.16). Lastly, a regression was conducted examining whether those who perceive shame and stigma around STIs get tested for STIs. The overall model was significant (*F*(5,184) = 11.33, *p* < 0.001, adjusted *R*^2^ = 0.22, *R*^2^ change = 0.02), as was the standardized coefficients, (*ß* = 0.16, *p* = 0.02). To explore whether these relationships were a partially mediated model, I ran a Sobel test [47], which barely reached significance (*z* = −1.59, *p* = 0.10).

To explore the second half of RQ4, a similar set of tests was conducted using the same first regression (STI knowledge and shame and stigma) with shame and stigma as the independent variable in Block 2, placing communication about STIs with parent as the dependent variable, and then also communication with partners. The Sobel tests were significant, both with parental communication about STIs (*z* = 1.89, *p* = 0.05), and partner communication (*z* = 1.95, *p* = 0.05). The results to the individual regression analyses can be found in Table 3, with change in *R*^2^ indicating the amount of variance each variable accounted for.

## 5. Discussion

The purpose of this study was to examine EAs’ perception of shame and stigma surrounding STI disclosure and communication. Results suggest that although the EAs in this study may not feel immense shame and stigma about STIs (e.g., getting tested or treated for) specifically—the scale was relatively normally distributed—they would feel shame and stigma in some situations. Just thinking that some members of their community knew they got tested for STIs, or found out the results of said test, left EAs feeling as though they would likely be treated differently. However, this study did not indicate EAs’ concern about such treatment (e.g., better or worse). That is, we did not measure if EAs would care if they were treated worse, or if it would impact them in any way. This study also found that if EAs had to actually engage in a conversation about STIs and/or disclose a hypothetical STI status they would likely experience some discomfort. This is the case when EAs disclose to parents (for social/financial/informational/health care support), monogamous sexual partners, and casual sexual partners. 

Interestingly, disclosing an STI status to a monogamous sexual partner yields less felt shame and stigma as compared to disclosing to either parents or a casual sexual partner. Although there is little research on disclosure of STI statuses to monogamous versus casual sexual partners, plenty of health websites (e.g., WebMD, HealthLine) provide guidance on when and how to reveal your STI status to a sexual partner. For example, WebMD tells people to be in a private space, straightforward, calm, and sincere. The site also states to stray away from disclosing “in the midst of a passionate embrace,” and to plan ahead [48]. Planning and potentially having a script ready makes theoretical sense, however, for the life of an EA amidst the hook-up culture, such preemptive thought may be more difficult. This may be one reason why disclosing to a monogamous romantic partner carries less perceived stigma with it. That is, EAs can think and plan and develop a script to sit down with their partner and disclose their status before engaging in sexual activity, as compared to casual sexual partners in the hook-up culture that may involve alcohol, drugs, and more impromptu sexual encounters. Monogamous partners also have more trust, security, and stability, allowing people to feel supported [49]. This becomes even more important for those with a chronic STIs to disclose [49]. Thus, it makes sense that if EAs feel supported and secure in their relationships, they perceive less stigma at disclosing an STI. It is also likely that EAs would be expected to disclose STIs early on in a monogamous relationship, questioning just how much trust is present. However, if EAs see a future monogamous romantic relationship with this person, although the trust would not be at the same level as a well-established partnership, it is likely they see that trust grow, and disclosing an STI status could help build the trust and intimacy within the relationship, just as disclosing any private information does [50].

We should note, much discussion of monogamous relationships does so in the traditional, binary and ideological sense. Thus, it is imperative to replicate this study allowing participants to identify what monogamy meant to them, whether it was long-term and binary, or some agreed-upon relational formation (e.g., polyamorous) that may still allow for trust and security but may not be “traditional”. It may also move the field forward to explore these ideas of shame and stigma surrounding STIs and sex communication beyond casual and monogamous sex, examining outcomes and perception of felt stigma in consensually non-monogamous partners. 

With other sexual relationships, people tend to manage their self-relevant information in order to make positive impressions on others [51]. Thus, although sharing and exchanging personal information is imperative to develop relationships, at an early stage, people may be highly cognizant of the impression they give off [52], especially within emerging adulthood and college, when coolness is an imperative factor of everyday life [53]. Therefore, disclosing an STI status to casual sexual partners, although an important part of sex communication and sexual interactions, makes impression management more difficult. It also makes sense that disclosing an STI status to someone who is basically a stranger, with a lack of trust and security within the relationship, comes with felt stigma and shame for EAs. Future studies should be more specific with types of causal sexual partners, as this study did allow participants to think of casual sexual partners as whatever they perceive them to be. For some, casual sexual partners can be friends with benefits or first-time hook ups with good friends, in addition to almost strangers. Thus, our explanation of having less trust within casual sexual interactions may not always be the case. 

This study also shed light on how shame and stigma impacted the overall communicative process of talking about sex and STIs with parents and sexual partners. Shame and stigma were significantly and negatively associated with parental communication. That is, the more shame and stigma EAs experienced or perceived around STIs, the less likely they were to talk about STIs and sex with their parents, even when in need of social support of varying nature (e.g., financial, informational, emotional). There was a similar relationship (i.e., negative and significant) between STI-related shame and stigma and partner communication. As this is a regression analysis, it could be argued that the relationship could go either way, since a decrease in parental communication about sex and STIs yields higher perceived shame and stigma surrounding STIs. There were no relationships, however, between shame and stigma and whether they have, or would, disclose an STI to parents or sexual partners. 

Lastly, the relationship between STI knowledge and getting tested for STIs seemed to be mediated by shame and stigma. Similarly, the relationships between shame and stigma and getting tested were partially mediated by communication with parents, and in another model, mediated by communication with partners about STIs and sex. That is, an increase in knowledge about STIs decreased STI-related shame and stigma, which increased the likelihood to get tested for STIs. Additionally, as STI-related shame and stigma increased, communication with parents or partners about STIs or safe sex decreased, which decreased the likelihood of getting tested for STIs. 

This finding is novel to the field as past research has primarily only argued that STI-related shame and stigma, alone, were enough to prevent getting, or find the results of, STI tests [23,54]. This study also revealed this to be true, (*F*(5,184) = 11.33, *p* < 0.001, adjusted *R*^2^ = 0.22, *R*^2^ *change* = 0.02; *ß* = 0.16, *p* = 0.02), however, the relationships were partially, but significantly, mediated by parental and partner communication, with highly significant pathways and a significant overall Sobel test statistic. In other words, having conversations about STIs, STI risks, and safe sex practices with parents can increase the likelihood of getting STI tests when sexually active. Having partner communication about sex, safe sex, STIs, and STI risks can also increase the likelihood EAs get tested for STIs, which is imperative with the nature of the hook-up culture. 

The present research is a useful first step in identifying theoretically valuable understandings about how shame and stigma surrounding STIs have shifted within the hook-up culture, which impacts EAs’ disclosure and communicative processes. 

### Limitations and Future Directions

Although this study aims to make unique contributions to communication and health scholarship, there are limitations. First, this study relied on an undergraduate student research pool. Although this pool includes the student body of a large Southwestern university that is relatively diverse in nature, this sample is rather homogenous. Most participants identified as White (Non-Hispanic; 62%) and female (80.7%), and they averaged 20.1 years of age (*SD* = 1.29), with few participants under 19 years old (*n* = 39) or over 22 (*n* = 11). Increasing the variance of the sample would allow for a more inclusive understanding of the communicative processes surrounding STI shame, stigma, and disclosure. Furthermore, having a more diverse population may allow for effects that differ due to differences in culture surrounding sex. 

Future research may benefit from adding some participation requirements, as well as measures that were not considered prior to examining participants’ responses during the analyses. First, it may be of interest to conduct a replication of this study with the limitation of participants who have experienced STI testing, or an STI diagnosis. Including those who have been diagnosed and actually experienced the process of disclosing may allow for more thorough within-group comparisons and a different perspective on the communicative processes. Comparing a replication of this study to the results provided here would allow us to see the differences in disclosure processes, as well as how diagnosed individuals perceive their peers’ perceptions of STIs, communication about sex, and the process of disclosing. The timing or context of the contraction of the STI may also be an important variable to consider. That is, many participants remarked that if they had cheated and contracted the STI, they would not expect their partner to support them, whereas if they had contracted an STI prior to their current partner, they would expect more support. Similarly, research may want to consider separately disclosure of the STI status to the partner that potentially gave the participant an STI, as well as the current partner, if they are in fact different individuals. Lastly, future research should dig into the relationships between perceived shame and stigma surrounding STIs and the ability to have safe sex conversations with both monogamous romantic and casual sexual partners. That is, exploring how EAs weigh risking getting an STI—one that may just go away on its own and show no symptoms—from new sexual partners and the stigma and complications (e.g., showing lack of trust, love, or even subtly telling a partner you have an STI) of condom negotiation. 

Lastly, it is important to note that the survey distributed did not specify STI type in the verbiage of the questions. Communication (e.g., uncertainty, information management) and disclosure theory has long argued that how someone assesses the problem or issue impacts how they then proceed. The health disclosure decision-making model [55] argues that we assess five factors surrounding our health-related issue: stigma surrounding it, preparation required to deal with the health diagnosis, actual prognosis and outcomes, symptoms related to the diagnosis, and its relevance to others. In the case of STIs, it could be argued each of these is important to consider when exploring whether EAs would disclose their STI status to their parent or a sexual partner. Potentially, most importantly, the actual prognosis and symptoms. That is, if an STI is chronic, EAs may feel a need to disclose their STI to others, especially if it impacts the other person by way of sexual contact or that they will need to provide support down the road. With a chronic STI, infected individuals may weigh the benefit of disclosing information to their parents more than the risks associated with having that type of conversation. A chronic STI is also likely to impact everyday life when it comes to self-esteem as it relates to sexual identity, desirability, and ability to form strong intimate relationships in the future. Chronic STIs may also require more social support from parents, like emotional support to cope with any psychological issues that may arise, or financial support for consistent and life-long treatments. However, when the majority of STIs are treatable and/or show no symptoms [2], even when EAs require treatment, these often dissipate as the infection ceases to exist. Thus, EAs may be able to manage their infection independently without the need to disclose to a parent or any sexual partners. Therefore, future research should indicate type of STI, chronic or acute, when exploring perceived stigma and shame. With this research setting the groundwork to better understand EAs’ perception of stigma and shame surrounding STIs, future research would benefit from exploring the other four aspects of information people assess when deciding to disclose personal health information [55]. Doing so could also shed light on EAs’ perception on STI prevalence and treatments available, and how they weigh the costs and benefits of disclosing any potential STIs with condom negotiation conversations, as well as STI disclosure.

## 6. Conclusions

This study provides insight into the way EAs are experiencing STIs, including those who have not been diagnosed with an infection. Past research provides evidence for the idea that shame and stigma surrounding STIs can delay treatment for people of all ages [23,54,56,57], potentially decreasing overall physical health, thus increasing the likelihood of spreading to others. However, if we continue to conceptualize and operationalize shame and stigma surrounding STIs using the traditional definitions, we may be missing out on a major component that impacts individuals’ engagement in healthy sexual behaviors, including condom negotiation conversations with potential sexual partners and support-seeking behaviors after a diagnosis. Furthermore, the operationalization of the traditional measure is quite strongly worded (e.g., “I have poor morals,” or “getting an STI would be embarrassing”). Thus, many of these EAs may have some shame related to STIs, but not to that extent, considering the low amount of variance of this measure. This study therefore helps to extend our understanding and conceptualization of what the stigma surrounding STIs is and how it may differ for EAs and therefore impact the likelihood of their engagement in healthy sexual behaviors. The results of this study can also help to thoroughly develop the operationalization used for shame and stigma about STIs (i.e., using items worded less strongly and creating encompassing processes surrounding STIs beyond treatment and health care providers), allowing researchers to more fully understand the experience, differences that varying ages may face, and motivations behind sexual health conversations and seeking the support necessary to get tested and adhere to treatment regiments. 

The results of this study provide an opportunity to develop stronger, theory-based health interventions targeting EAs and their sexual behaviors, safe sex practices, and STI testing. Specifically, knowing that EAs’ perceived shame and stigma surrounding STIs may be how health interventions encourage this demographic to get tested and treated. Rather than arguing issues currently used as costs (e.g., general prevalence, long term effects if gone untreated), health interventions can flip the rhetoric using these now non-issues as benefits of getting tested. That is, due to prevalence, getting tested and treated are easier and more private. This study does show that EAs still think many people in their lives may treat them differently if an STI status was disclosed. Therefore, knowing the importance of impression management during emerging adulthood may be another way STI testing and STI communication campaigns can encourage EAs to get tested in the first place, allowing EAs to avoid the uncomfortable conversation of disclosing an STI to their romantic partner.

Moreover, this study provided insight into the differences in shame and stigma EAs feel concerning STIs. As sexual exploration is commonplace [30] for EAs and within the hook-up culture [11] it is important to consider the communicative processes surrounding EAs’ perception of STIs. Seemingly they are willing to get tested and find out the results, however, taking the step to engage in conversation about STIs is more difficult. Thus, although it is important for EAs to get tested and get treated, talking about STIs can help to prevent spread and contraction [23]. The results of this study in conjunction with past research argue for more targeted health-promotion tactics that also encourage talk, the starting point towards increasing condom usage, consent, and healthier sexual relationships within the EA population [12].

Understanding how EAs perceive communication about STIs with their parents is another component to consider when developing interventions and programs. Conceptualizing this type of talk is also important when we consider the role health insurance, and the Affordable Care Act, has on the relationship between parents and EAs. That is, many individuals may remain on their parents’ health insurance until they are 26 years old. Any services they receive, including STI testing, will be noted on their parents’ health insurance itemized bills, disclosing EAs’ private information to them. Health-promotion initiatives may therefore want to focus on assisting EAs in becoming more assertive about engaging in communication about sex with their parents, especially as it pertains to safe sex practices and health care surrounding sex (e.g., STI testing). Such conversations with parents may guide EAs to practice safe sex behaviors [12]; furthermore, having positive, open discussions about sex can assist the EA in building relationships and increasing sexual confidence [58]. Thus, the EA may become more assertive about engaging in communication about sex with romantic partners, thus reducing risky behavior enactment and the consequences associated with those behaviors.

Clearly, this project only begins to scratch the surface of our understanding of how shame and stigma towards STIs has changed, especially among EAs who are part of the “hook-up culture.” Though people used to experience shame and stigma when getting tested and finding out the results [23], hence it became the traditional measure primarily used in research [8], this study suggests we may need to focus more on the disclosure process. Disclosing an STI status not only allows EAs to receive social support in its various forms from parents and romantic partners, but also allows EAs to initiate conversations with partners about safe sex practices.

## Figures and Tables

**Table 1:** First Person Independent and Dependent Variables Correlation Matrix.

Variable	*M*	*SD*	1	2	3	4	5	6	7	8	9	10	11	12	
1. Health Literacy	4.92	1.63													
2. STI Know ^a^	8.17	1.63	0.38 ***												
3. Percep of STI Know	5.40	1.07	0.13 *	0.20 **	**0.86** ^d^										
4. Percep of Support	3.77	0.65	0.71 **	0.50	0.22	**0.71**									
5. Disclose Parents	3.78	0.80	0.28 **	0.21 *	0.18	0.09	**0.61**								
6. Disclose Monog ^b^	2.86	1.07	−0.46	−0.64	−0.37	0.00	−0.19	**0.81**							
7. Disclose Casual	3.17	0.92	−0.04	0.05	0.18	0.45	0.55 **	−0.29	**0.76**						
8. Shame and Stigma	2.57	0.76	−0.16 ***	−0.17 *	−0.14 *	−0.73 **	−0.06	0.88 **	−0.00	**0.90**					
9. SS ^c^ Disclose Parents	3.40	1.04	−0.06	−0.00	−0.09	−0.57 *	0.20 *	0.81 *	0.07	0.50 ***	**0.92**				
10. SS Disclose Monog	3.28	0.90	−0.07	−0.04	−0.03	−0.53	0.20 *	0.48	0.31 *	0.48 ***	0.51 ***	**0.91**			
11. SS Disclose Casual	3.43	0.90	−0.01	−0.02	−0.12	−0.47	0.21 *	0.71	0.06	0.43 ***	0.53 ***	0.60 ***	**0.91**		
12. Comm STI—Parent	2.80	1.12	−0.01	0.08	0.26 ***	0.24	−0.18	−0.25	0.03	−0.16 ***	−0.32 ***	−0.07	−0.09 *	**0.94**	
13. Comm STI—Partner	3.09	0.84	0.04	0.13 *	0.22 ***	−0.04	0.21 *	0.65	0.02	−0.21 ***	−0.09	−0.14 **	−0.08	0.34 ***	**0.90**

^a^ Knowledge; ^b^ Monogamous Partner; ^c^ Shame and Stigma; ^d^ Diagonal bold is Cronbach’s Alpha, Health literacy and STI knowledge are actual measures of knowledge, correct responses summed, and do not have reliability measures; * *p* < 0.05, ** *p* < 0.01, *** *p* < 0.001.

**Table 2:** Pairwise Comparisons of Shame and Stigma of Disclosure—One-Way Repeated-Measures ANOVA.

Disclosure to	Mean Difference	Standard Error		Significance (*p*)	Confidence Interval	
					Lower Bound	Upper Bound
Parents	Monogamous	0.12	0.05	0.02 *	0.01	0.23
	Casual	−0.03	0.05	1.0	−0.13	0.08
Monogamous	Parents	−0.12	0.05	0.02 *	−0.23	−0.01
	Casual	−0.15	0.04	0.00 ***	−0.24	−0.06
Casual	Parents	0.03	0.05	1.0	−0.08	0.13
	Monogamous	0.15	0.04	0.00 ***	0.06	0.24

* *p* < 0.05, *** *p* < 0.001.

**Table 3:** Multiple Regressions between Variables.

DV	IV	*F*	*Adj R* ^2^	Δ*R*^2^	*p*	*ß*	*p*
**Mediation #1**							
Knowledge	Shame/Stigma	2.7	0.09	0.05	0.03 *	−0.22	0.04 *
Shame/Stigma	Get Tested	11.33	0.22	0.02	<0.001 ***	0.16	0.02 *
**Mediation #2**							
Shame/Stigma	Parent Comm	2.43	0.04	0.03	0.04 *	−0.16	0.03 *
Parent Comm	Get Tested	13.13	0.24	0.05	<0.001 ***	−0.23	<0.001 ***
**Mediation #3**							
Shame/Stigma	Partner Comm	3.24	0.06	0.03	0.008 **	−0.19	0.01 *
Partner Comm	Get Tested	12.05	0.23	0.04	<0.001 ***	−0.19	0.004 **

* *p* < 0.05, ** *p* < 0.01, and *** *p* < 0.001.

## Data Availability

The data presented in this study are available on request from the corresponding author. The data are not publicly available due to IRB protocol at time of approval.
